# The Tumor Microenvironment in Neuroblastoma: New Players, New Mechanisms of Interaction and New Perspectives

**DOI:** 10.3390/cancers12102912

**Published:** 2020-10-10

**Authors:** Laurence Blavier, Ren-Ming Yang, Yves A. DeClerck

**Affiliations:** 1 The Saban Research Institute, Children’s Hospital Los Angeles, Los Angeles, CA 90027, USA; lsarte@chla.usc.edu (L.B.); rmyang@chla.usc.edu (R.-M.Y.); 2 Division of Hematology, Oncology and Blood and Bone Marrow Transplantation, Department of Pediatrics, Keck School of Medicine, University of Southern California, Los Angeles, CA 90033, USA; 3 Department of Biochemistry and Molecular Medicine, Keck School of Medicine, University of Southern California, Los Angeles, CA 90033, USA

**Keywords:** neuroblastoma, tumor microenvironment, cancer-associated fibroblasts, MYCN, extracellular vesicles, microRNA

## Abstract

**Simple Summary:**

Neuroblastoma is the second most common solid tumor in children. Our understanding of the contribution of genetic factors (seed) that contribute to neuroblastoma progression has substantially improved in the last 2 decades but the contribution of the tumor microenvironment (TME, soil) is the subject of more recent attention. Here we highlight recent studies pointing to novel mechanisms by which the TME affects neuroblastoma progression. Cancer-associated fibroblasts are present in neuroblastoma tumors and contribute to escape from chemotherapy and immunotherapy. Extracellular vesicles and regulatory micro-RNAs they contain, serve as shuttle mechanisms between neuroblastoma cells and stromal cells. The TME landscape of neuroblastoma differs between MYCN amplified and MYCN-non amplified tumors with the former being “cold” and the latter “hot” and rich in inflammatory cells. These recent observations will have a significant impact on our ability to design precise clinical trials that integrate information on the neuroblastoma cells and on their TME.

**Abstract:**

The contribution of the tumor microenvironment (TME) to cancer progression has been well recognized in recent decades. As cancer therapeutic strategies are increasingly precise and include immunotherapies, knowledge of the nature and function of the TME in a tumor becomes essential. Our understanding of the TME in neuroblastoma (NB), the second most common solid tumor in children, has significantly progressed from an initial focus on its Schwannian component to a better awareness of its complex nature, which includes not only immune but also non-immune cells such as cancer-associated fibroblasts (CAFs), the contribution of which to inflammation and interaction with tumor-associated macrophages (TAMs) is now recognized. Recent studies on the TME landscape of NB tumors also suggest significant differences between MYCN-amplified (MYCN-A) and non-amplified (MYCN-NA) tumors, in their content in stromal and inflammatory cells and their immunosuppressive activity. Extracellular vesicles (EVs) released by cells in the TME and microRNAs (miRs) present in their cargo could play important roles in the communication between NB cells and the TME. This review article discusses these new aspects of the TME in NB and the impact that information on the TME landscape in NB will have in the design of precise, biomarker-integrated clinical trials.

## 1. Introduction

Non-malignant cells in the tumor microenvironment (TME) are not innocent bystanders and can positively or negatively affect the growth, survival and metastatic potential of tumor cells [1]. Innate and adaptative immune cells such as tumor-associated macrophages (TAMs), dendritic cells (DC), natural killer (NK) cells and NKT cells and T and B lymphocytes have been the focus of much attention recently, with new developments in immunotherapy. However non-immune cells such as endothelial cells (ECs), pericytes, Schwann cells (SCs), and, more recently described, cancer-associated fibroblasts (CAFs) also contribute to the TME in neuroblastoma (NB) tumors. Our understanding of the nature and the function of the TME in NB has significantly progressed in recent years. The presence of SCs in NB tumors was recognized in the early 1980s [2] as a marker of favorable outcome [3], and the contribution of ECs and tumor-derived vascular endothelial cell growth factor (VEGF) to NB progression was reported in the late 1990s [4]. The presence of α-smooth muscle actin (α-SMA)-positive CAFs has been more recently reported [5,6]. In recent years, we have gained a significantly better understanding of the mechanisms by which non-immune stromal cells in the TME affect the behavior of tumor cells, and of the multiple ways tumor cells and TME cells communicate. At the same time, there have been suggestions that amplification of the MYCN oncogene may affect the nature and function of the TME in NB tumors and that extracellular vesicles (EVs) represent a mechanism of communication between tumor cells and non-malignant cells in the TME. The focus of this review is to highlight these recent observations and to discuss their potentially important impact on our ability to develop new biomarker-guided therapeutic clinical trials for children with high-risk NB.

## 2. CAFs, New Players Cooperating with TAMs in NB Progression

The presence of CAFs in many solid tumors such as pancreatic, lung, colon and breast cancers has been recognized for many years. They contribute to a desmoplastic reaction, increase tumor tissue stiffness and promote the proliferation and invasion of cancer cells [7,8]. CAFs represent a heterogeneous population of mesenchymal cells expressing a variety of markers such as fibroblast specific protein (FSP)-1 (aka S100A4), fibroblast activation protein-α (FAP)-α, (aka dipeptidyl peptidase IV), α-SMA, platelet-derived growth factor receptor (PDGFR)-β, and β1 integrin (CD29) in addition to other proteins such as thrombospondin-1, tenascin-C, stromelysin, desmin-1, and VEGF-A [9,10]. In contrast to normal resting fibroblasts, CAFs rapidly proliferate, secrete an abundance of extracellular matrix (ECM) proteins including collagen and fibronectin, and are migratory. When activated by the presence of tumor cells, these fibroblasts become “educated” and secrete inflammatory proteins [11,12,13,14]. Diverse mechanisms of CAF activation have been identified, including TGF-β, inflammatory signals such as interleukin (IL)-6, IL-8, or tumor necrosis factor (TNF), DNA damage, physiological stress, or change in ECM [8,11,15]. It has been suggested that CAFs originate from a variety of cell types including resident fibroblasts, ECs, bone marrow-derived mesenchymal stromal cells (MSCs) and even induced pluripotent stem cells (iPSC) [11,12,13,14,16,17,18,19,20].

The presence of CAFs in NB tumors was first recognized by S. L. Cohn’s group, who reported the presence of α-SMA-positive non-pericytic cells in NB tumors and reported that the presence of these cells was associated with an increase in microvascular proliferation, a decrease in the presence of SCs, and with an overall poorer clinical outcome [5], an observation suggesting that CAFs could contribute to a TME favorable to NB cell growth. Our laboratory has since reported similar observations identifying CAFs that share phenotypic and functional characteristics of bone marrow-derived MSCs, isolated from fresh NB tumors. These stromal cells express several markers of activated CAFs such as FAP-α and FSP-1, as well as markers of MSCs such as CD105 (aka endoglin), CD90 (aka Thy-1) and CD73 (aka 5′-nucleotidase) and, like MSCs, maintain an ability to differentiate into osteoblasts, adipocytes or chondrocytes [6]. These cells, designated CAFs-MSCs, promote NB cell growth and survival, and enhance resistance to chemotherapy by being a source of multiple inflammatory cytokines and chemokines such as IL-6, IL-8, VEGF-A, chemokine C-C motif ligand (CCL)-2 [aka monocyte chemoattractant protein (MCP) 1] and C-X-C motif Ligand (CXCL)-12 [aka stromal-derived factor (SDF)-1] that target NB cells and stromal cells in the TME. These cytokines and chemokines activate signal transducer and activator of transcription (STAT)-3 and extracellular regulated kinase (ERK 1/2)-mediated signaling in NB cells that stimulate their proliferation, survival and drug resistance [21]. CXCL-12, produced by bone marrow-derived MSCs, promotes the homing of C-X-C motif receptor 4 (CXCR4)-expressing tumor cells to the metastatic bone marrow niche [22,23]. CAF-derived cytokines not only target NB cells but also non-malignant cells in the TME. IL-6 activates osteoclasts in the bone marrow that promote the formation of osteolytic metastasis [24]. VEGF-A and IL-8 stimulate angiogenesis and CCL2 is a chemoattractant for monocytes, macrophages and invariant NKT (iNKT) and NKT cells [25,26]. CAFs and MSCs are also a source of transforming growth factor (TGF)-β, which has a potent immunosuppressive activity on NK cells and T cells and promotes immune escape [27]. MSCs protect NB cells from H_2_O_2_-induced oxidative stress by their capacity to produce and activate antioxidant enzymes and to downregulate superoxide dismutase [28].

In NB tumors, CAFs are often present in close proximity of TAMs, and transcriptomic analysis of multiple NB datasets has shown a direct correlation between the expression of CD163 (a marker of M2 polarized macrophages) and FAP-α expression [6,29]. TAMs enhance CAF-induced STAT-3 activation in NB cells by being a source of the soluble agonistic receptor for IL-6 (sIL-6R) [21]. There is a reciprocal functional relationship between CAFs and TAMs. TAMs enhance not only the invasive abilities of CAFs but also their proliferation [28]. On their side, CAFs are a source of CCL2 that chemoattracts TAMs and a source of microsomal prostaglandin E synthase-1 (mPGES-1), the enzyme responsible for the production of PGE2 which enhances the M2 polarization of TAMs. Accordingly, inhibition of mPGES-1 in transgenic TH-MYCN mice resulted in a decrease in angiogenesis and tumor growth, and an increase in M1 macrophage polarization [30].

Altogether these data demonstrate that CAFs and their precursor cells, MSCs, have a pro-tumorigenic activity in NB. However, as the majority of these studies have been completed in vitro or in animal experiments, our understanding of their contribution to the progression and response to therapies in patients with NB tumors remains limited and will require a better knowledge of the TME landscape in NB tumors not only at the time of diagnosis but also at the time of recurrence.

## 3. Hot and Cold NB Tumors: Is There a Role for MYCN?

In recent years, several studies examining the landscape of the TME in NB tumors have been published. Because they have used a variety of methodologies, ranging from gene expression analysis of large datasets [31,32] to histology-based immunohistochemistry (IHC) in human xenotransplanted tumors and patient-derived tumors, they have sometimes generated conflicting data. However, a review of the recent literature described here suggests that there are significant differences in the landscape of the TME between MYCN-A and MYCN-NA tumors in regard to the presence of stromal and immune cells (Table 1). 

### 3.1. Stromal Cells

#### 3.1.1. The Schwannian TME in NB

The presence of SCs in NB tumors defines a “stroma-rich” group of tumors associated with a favorable outcome [33]. The origin of these cells remains controversial but there is genetic evidence that SCs either represent a reactive population of normal cells or originate from NB cells that have differentiated [42,43]. These SCs exhibit amplification of *MYCN* but typically have low levels of *MYCN* mRNA and protein expression [44]. MYCN promotes neural differentiation vs. SC differentiation in sympathoadrenal precursor cells [45]. High-risk MYCN-A and MYCN-NA tumors are noticeably poor in SCs and SC-rich NB tumors rarely exhibit MYCN [33]. SCs contribute to a TME that inhibits NB tumor growth by being a source of anti-angiogenic factors [46,47]. 

#### 3.1.2. MYCN-A Tumors are More Vascularized

NB tumors develop their vasculature in part through the production of VEGF-A controlled by growth factors such as insulin-like growth factor-1 (IGF-1), epidermal growth factor (EGF), or platelet-derived growth factor (PDGF) and by hypoxia. It has been shown that IGF-1 induced the production of hypoxia-inducible factor-1α (HIF-1α) and consequently VEGF mRNA transcription in NB cell lines, which effects could be blocked by Topotecan [48]. There are also multiple experimental evidences that MYCN plays an important active role in this aspect. The expression of HIF-1α is higher in MYCN-A tumors and MYCN can override HIF-1α inhibition. HIF-1α and MYCN are also essential for the Warburg effect (aerobic glycolysis) by their ability to activate the transcription of multiple glycolytic genes such as phosphoglycerate kinase 1 (PGK1), hexokinase 2 (HK2) and lactate dehydrogenase A (LDHA) that contribute to aerobic glycolysis versus oxidative phosphorylation as the source of energy and ATP [49]. HIF-1α depletion downregulates these three gene expressions at normoxia and inhibition of MYCN and HIF-1α also additively downregulates HK2 and LDHA expression [49]. These observations suggest that both MYCN and low levels of HIF-1α cooperatively contribute to the Warburg effect via regulation of glycolytic genes under normoxia. Under hypoxia however, stabilized HIF-1α, together with high levels of MYCN protein, increase glucose uptake with concomitant lactate production. HIF-1α expression is enhanced by factors that regulate the expression of MYCN or regulate its nuclear translocation, such as aurora kinase A (AURKA) [50]. NB cells also drive angiogenesis through the secretion of PDGF and fibroblast growth factors (FGF), the expression of which correlates with the *MYCN* status and the aggressiveness of the tumor [34,51,52]. MYCN expression in NB cells alters the angiogenic balance by downregulating EC growth inhibitors such as leukemia inhibitory factor (LIF), IL-6 and Activin A, but leaving the expression of the stimulators unaffected [53,54]. In MYCN-A tumors, ECs covered with pericytes can originate from tumor cells and this vasculomimicry contributes up to 70% of the tumor vasculature [55]. All these studies point to an active contribution of MYCN to tumor vascularization. Accordingly, analyses of patient-derived tumors revealed higher levels of tumor vascularization in MYCN-A tumors than in MYCN-NA tumors [34]. MYCN-A tumors have a higher expression of αvβ3 integrin in their ECs, which stimulates EC attachment to vitronectin and migration, and phosphoinositide 3 kinase (PI3K) signaling-dependent proliferation and survival [35]. The presence of pericytes in the vasculature of SC-poor tumors, and the contribution of matrix metalloproteinase (MMP)-9 to their recruitment in the perivascular space and to the integrity of the tumor vasculature, has been reported but the contribution of MYCN to this process has not been determined [56,57]. There is also suggestion that ALK promotes angiogenesis via VEGF, as ALK knockdown via siRNA was associated with marked reductions in VEGF secretion, blood vessel density, and MMP expression in mice [58].

#### 3.1.3. MSCs and CAFs in NB Tumors 

There have very been limited reports comparing the presence of MSCs and CAFs in MYCN-A and MYCN-NA NB tumors. In a study of 41 NB tumors analyzed by IHC (7 MYCN-A and 34 MYCN-NA), a higher presence of α-SMA-positive, non-pericytic (h-Caldesmon-negative) CAFs in MYCN-A tumors vs. MYCN-NA tumors, was reported [29]. A transcriptomic analysis of four datasets including the TARGET database suggests that MYCN-A tumors contain more stromal cells such as osteoblasts (CD44+ CDH1+), which originate from bone marrow derived MSCs under the influence of VEGF [59], and smooth muscle cells (α-SMA) but not fibroblasts (FSP1) and ECs when compared with MYCN-NA tumors [32]. As previously mentioned, α-SMA-positive non-pericyte CAFs are more abundant in SC-poor tumors [5].

### 3.2. Immune Cells 

It is well known that MYCN causes downregulation of major histocompatibility complex (MHC) class I antigen expression in NB that contributes to tumor antigen presentation and is necessary for the initiation of an immune attack [60,61]. This lack of antigen expression—with the notable exception of disialoganglioside (GD)-2—in NB has been responsible for the well-recognized ability of MYCN-A tumors to escape a cytotoxic T cell (CTL) and interferon-mediated immune response [62]. However, the contribution of MYCN to the recruitment of immune cells into the tumor and to the composition of the tumor immune microenvironment (TIME) has only begun to be explored. 

A transcriptomic analysis of the TARGET dataset using the CYBERSORT algorithm and validated by IHC of NB tumors identified a decrease in six types of immune cells (CD8+T cells, CD4+T cells, B cells, macrophages, DCs, and NK cells) in MYCN-A tumors when compared to MYCN-NA tumors [36]. Another transcriptomic analysis of 4 gene expression datasets indicated a higher presence of myeloid and lymphoid cells (DCs, Th2, CD8+T cells, NKT cells, macrophages) in MYCN-NA tumors vs. MYCN-A tumors [32]. Examining a series of 84 NB tumors by IHC for the presence of CD3+, CD4+ and CD8+ T cells and defining an immunoscore, Mina et al. found that infiltrating T cells have a good prognostic value independent of the current indicators used for NB staging including MYCN-A, but they reported the presence of a defined subset of infiltrating T cells (high CD3+ or low CD3+ and high CD4+/CD8+ ratio) associated with a more favorable outcome in both MYCN-A and MYCN-NA tumors [37]. Deep RNA-seq on untreated NB tumors validated by tissue microarray (TMA), and T-cell receptor (TCR) sequencing on additional tumors revealed that MYCN-NA tumors had significantly higher cytotoxic tumor infiltrating lymphocytes (TIL) signatures compared with MYCN-A tumors [38]. The presence of regulatory T cells (Treg) in NB tumors has not been reported but *MYCN* amplification in NB tumors correlates with a higher number of CD4+/CD25hi/CD127-Treg cells in bone marrow (BM) and of CD4+/CD45R0+/CD49b+/LAG3+ type 1 regulatory T (Tr1) cells in peripheral blood (PB) of patients [39]. CD1d-restricted iNKT (also known as type 1 NKT cells that recognize lipids rather than peptides) and type 2 NKT cells have also been shown to be more abundant in MYCN-NA tumors rather than MYCN-A tumors. A reverse correlation between mRNA reflecting NKT cells and *MYCN*-mRNA was reported in 98 primary untreated NB tumors [59]. iNKT cells migrate toward NB cells in a CCL2-dependent manner, preferentially infiltrating MYCN-NA tumors that express CCL2 as MYCN downregulates CCL2 [25]. NK cells are also decreased in MYCN-A tumors. An analysis of TARGET transcriptomics data, not presently validated by IHC, indicates a decreased expression of NK genes in MYCN-A tumors [36]. There is an inverse correlation between MYCN expression and that of ligands for NK-cell-activating receptors and *MYCN* acts as an immunosuppressive oncogene in NB cells because MYCN protein negatively regulates the expression of ligands for the NKG2D and DNAM-1 (aka CD226) receptors that activate NK cells [40]. Two in silico transcriptomics data analyses indicated that B cells are rare or absent in MYCN-A tumors [32,36].

The contribution of MYCN to myeloid cells and macrophage tumor infiltration and polarization is less clear. The expression of colony stimulating factor receptor (CSF1-R), CD14 and CD68 in myeloid cells was examined by gene expression analysis in NB tumors and reported to be associated with a worse outcome, but possible differences between MYCN-A and MYCN-NA were not described [63].

In a gene expression analysis of 133 metastatic MYCN-NA tumors from children older than 18 months, validated by TMA for CD163 expression, Asgharzadeh et al. reported a high presence of M2 macrophages in these patients, suggesting an increase in polarization toward M2 in the absence of MYCN [41]. A more recent but also more limited analysis of 41 NB tumors by IHC suggested a higher presence of CD163+ macrophages in MYCN-A (*n* = 7) tumors than in MYCN-NA tumors [29]. Little is known of the role of granulocytes and mast cells in NB but an increased expression of mast cell-derived genes in MYCN-A tumors vs. MYCN-NA tumors has been reported [32].

**Figure 1:**
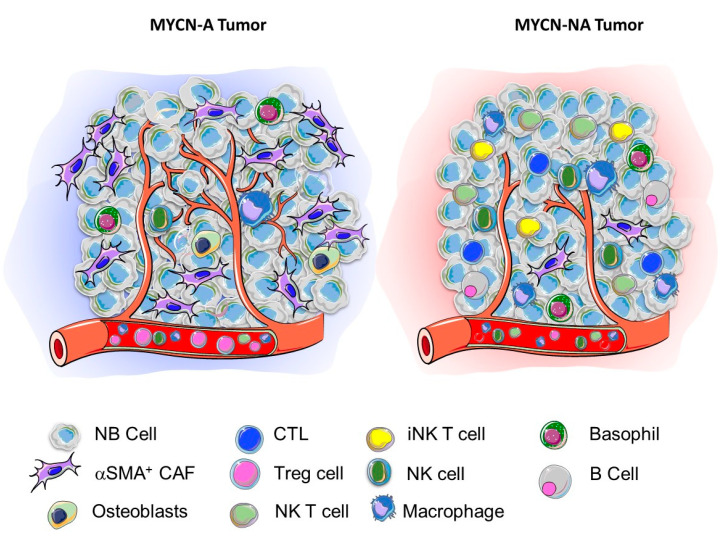
Contribution of MYCN to the TNME landscape in NB. Schematic representation of the TME landscape of a MYCN-A (cold) and a MYCN-NA (hot) tumor based on the review of the literature summarized in Table 1.

It is presently difficult to draw definite conclusions from these studies because of their limited number, the low number of tumor samples examined in some studies and the different methodologies used that spanned from regular histology, IHC, and TMA often restricted to the detection of a few markers, to in silico analysis of large transcriptomics datasets not always validated at the protein level. These studies are nevertheless informative allowing a picture of the landscape of the TME in NB to emerge. MYCN-A tumors are richer in stromal cells such as notably ECs, and α-SMA+ cells and poorer in immune cells such as CD4+ and CD8+ CTL, B cells, NK cells and NKT cells. In contrast, MYCN-NA tumors are less vascularized, contain fewer α-SMA+ cells and other stromal cells but are more infiltrated with innate and adaptative immune cells, in part through their higher expression of chemokines which are downregulated by MYCN. MYCN-A tumors therefore appear typically “cold” and immune exclusive, whereas MYCN-NA tumors seem inflamed or “hot” (Figure 1). These studies also raised the question of a potential role of MYC (aka cMYC), the expression of which is elevated in high-risk MYCN-NA tumors [64]. There is some recent in vitro and in vivo evidence that MYC controls the expression of the immune checkpoint molecule programmed death-ligand 1 (PD-L1) in NB cells and that an abundance of PD-L1 transcript correlates with MYC expression in NB tumors [40]. MYC could thus contribute to the creation of an immunosuppressive environment in a “hot” MYCN-NA tumor. A validation of this picture is however awaiting a more precise analysis of the TME landscape of NB tumors, including not only untreated primary tumors but also recurrent/refractory tumors with more sophisticated techniques such as multiplex IHC, imaging mass cytometry (IMC) and single cell analysis. Single-cell RNA-sequencing (scRNASeq) analysis have been reported to characterize the TME landscape of breast cancer [65], melanoma [66] and prostate cancer [67] but such studies have not yet been reported in NB. An online non-peer-reviewed publication from the Children’s Cancer Institute Australia in Sydney provides scRNASeq data showing that malignant neuroblasts move between adrenergic and mesenchymal cell states via a novel state that they termed a “transitional” phenotype [68].

## 4. EVs and miRs, New Mechanisms of Communication Between NB Cells and TME Cells 

### 4.1. EVs, a Family of Vesicles Released by Cells Including NB Cells

The communication between NB cells and TME cells mainly occurs through contact-independent mechanisms and is mediated by soluble proteins such as growth factors, cytokines, and chemokines. However, there has been more recent evidence that other soluble factors such as EVs could have a role. The term “extracellular vesicles” or “EVs” has been agreed on by the International Society for Extracellular Vesicles (ISEV) as the consensus generic term for lipid bilayer-delimited particles released from the cell [69]. EVs represent a large family of vesicles released by all cells, with a size ranging from 20 nm to 5 µm in diameter. Based on their size, subcellular origin, and composition, they can be classified into three groups: (1) exosomes, originating from late endosomes (aka multivesicular bodies-MVB), with a size ranging from 50 to 120 nm and sometimes divided into two subpopulations of small (Exo-S, 60–80 nm) and large (Exo-L, 90–120 nm) exosomes; (2) microvesicles with a size of 200–400 nm issued from the budding of the plasma membrane during cell stress; and (3) large >1 µm diameter apoptotic bodies, released by dying cells [70,71].

Exosomes differ from other EVs by their biogenesis and the presence of specific proteins. They are enriched in the three tetraspanins CD9, CD63 and CD81, heat shock proteins (HSP70 and HSP90), MHC class I and class II proteins, proteins of the endosomal sorting complex required for transport (ESCRT) that drive the formation of MVB and associated proteins such as TSG101 and Alix. They have a unique biogenesis as they derive from intraluminal vesicles (ILVs) formed by inward budding in MVB [69,72,73]. When MVB fuse with lysosomes, their cargo is degraded, but when they fuse with the plasma membrane, ILVs are released into the extracellular space as exosomes [74,75]. Exosomes contain a large variety of proteins, lipids, metabolites and nucleic acids including regulatory microRNAs (miRs), and act as bi-directional shuttles mediating the communication between tumor cells and TME cells, supporting the growth of the primary tumor and its progression to a metastatic stage [70,76]. In particular, tumor-derived exosomes have been shown to contribute to the pre-metastatic niche. They have an organotropism for metastatic organs mediated by integrins [77,78] and the intravenous injections of EVs in tumor-bearing mice enhances their metastatic potential in melanoma [79], pancreatic cancer [80], and in colon cancer [81]. Like all cancer cells, NB cells release EVs in the extracellular space. 

### 4.2. EVs Contain NB-Derived Proteins and Regulatory miRs 

In addition to exosomal markers proteins, the cargo of NB exosomes contains GD2 and proteins involved in tumor progression such as CD147 (basigin, aka extracellular matrix metalloproteinase inducer (EMMPRIN)), a transmembrane protein involved in invasion and metastasis, and CD276/B7-H3, an immune checkpoint protein that protects NB cells from attack by NK cells [82,83]. Comparative analysis of the proteomic cargo of exosomes isolated from NB cell lines revealed that some proteins constitute a common signature of those exosomes, while others are differentially expressed and reflect cellular behavior. Exosomes from cell lines derived from primary tumors (IMR32 and IGR-NB8) contain a higher level of proteins involved in ECM assembly and adhesion, as well as in neuronal development, whereas exosomes isolated from cell lines derived from bone marrow metastasis (SK-N-SH, SH-SY5Y, IGR-N91, SK-N-BE(2)-C, and LAN-1) contain proteins that are associated with cell survival, proliferation and motility [84]. Proteomic analysis performed on exosomes from two NB cell lines with different MYCN amplification status reflects their role in the aggressiveness of NB, as exosomes from MYCN-A SK-N-BE(2) cells are enriched in proteins involved in signal transduction, cell communication and transport, while exosomes from MYCN-NA SH-SY5Y are enriched in protein metabolism, cell growth, and maintenance and regulation of nucleic acid metabolism, suggesting that SK-N-BE2 cell-derived exosomes are enriched with proteins that could regulate various signaling pathways in the recipient cells [85]. More recently, a mass spectrometry-based proteomic profiling study of EVs isolated from plasma of NB patients, compared to healthy controls, has led to the identification of a NB specific signature including ferritin heavy chain (FHC), keratin, type I cytoskeletal 17 (KRT17), histone H3.3 (H3F3A), ATP binding cassette sub-family B member 9 (ABCB9), a disintegrin and metalloproteinase with thrombospondin motifs 13 (ADAMS13), CD14, erythrocyte membrane protein band 4.2 (EPB42), hepatocyte growth factor activator (HGFAC), keratin, type I cytoskeletal 13 (KRT13), and KRT8, all proteins related to cell proliferation and differentiation [86]. This type of analysis across multiple cancers led to the identification of tumor associated EV signatures, although it is still unclear what the role of these EV cargo proteins might be.

EVs are also rich in microRNAs (miRs), small non-coding RNA that regulate gene expression at post-transcriptional levels and control a variety of cellular functions [75]. The best-known mode of action of miRs is to prevent the translation of target mRNA into proteins however it has been shown that miRs can also increase the translation of the mRNA [87,88]. As miRs get transferred from cell to cell via EVs, they play an important role in cell communication in the TME [87]. The profile of miR content of EVs from MYCN-A NB cell lines (SK-N-BE(2)-C and Kelly) revealed the presence of several oncogenic miRs including miR-16, miR-125b, miR-21, miR-23a, miR-24, miR-25, miR-27b, miR-218, miR-320a, and miR-92a. A functional enrichment analysis using predicted mRNA target genes identified the aryl hydrocarbon receptor (AHR), the protein of which downregulates MYCN expression and promotes differentiation of NB cells, as a major mRNA target downregulated by these oncogenic miRs [79,89].

These observations raise the question of the function of the proteins and miRs present in the cargo of EVs in cells capturing these EVs through endocytosis, and whether the analysis of the protein and miR cargo of NB-derived EVs in the blood or other biological fluids of patients with NB reflects the tumor stage and the degree of aggressiveness of the tumor and could be used as a biomarkers. These questions are discussed below.

### 4.3. Function of EVs in NB Cell-TME Cell Communication 

Tumor-derived EVs can be captured by tumor cells (autocrine mechanism) or by stromal and immune cells in the proximal (paracrine) or distal (endocrine) TME [90]. Vice versa, EVs from TME cells can be captured by tumor cells. Our understanding of the mechanisms involved in these multiple interactions in NB is so far limited and primarily derived from in vitro co-culture experiments. Several studies have shown that EVs play a role in tumor cell to tumor cell communications, affecting the cells phenotype, their migratory potential, or conferring chemoresistance [85,91]. We also demonstrated that NB-derived EVs contribute to the communication between tumor cells and stromal cells, showing that exosomes derived from NB cells induced in vitro the production of pro-tumorigenic cytokines and chemokines such as IL-6, IL-8, VEGF, and CCL2 by MSCs [92]. 

In the TME, exosomal miRs are involved in a reciprocal interaction between NB cells and TME cells, triggering a pro-inflammatory response in monocytes which promotes the resistance of NB cells to chemotherapy [93]. Importantly, anticancer activities have been shown by exosomes derived from NK and activated NK (aNK) cells which contain miRs (miR186) and cytotoxic proteins (perforin, granulysin, and granzymes A and B) which, when captured by NB cells inhibit their growth and migration, prevent escape from NK cell-mediated cytotoxicity, and induce apoptosis [94,95]. 

A major limitation of the interpretation of these experiments is that they have been performed in vitro with EVs isolated from cells using a variety of methodologies. We also do not know how critical miRs present in tumor-derived EVs are in the induction of an inflammatory reaction in the TME. A better understanding of the role of EVs in NB cell-TME cell communication and in NB progression, metastasis, and therapeutic escape is awaiting carefully designed in vivo experiments and their validation in patient samples.

### 4.4. EVs and miRs as Liquid Biopsies 

The observation that the cargo of NB-derived EVs contains proteins and miRs that reflect the biology of the tumor cells raised the question whether the isolation of NB-derived EVs in the blood or other biological fluids in patients with NB could serve as diagnostic and prognostic biomarkers and indicators of therapeutic response. Recently, exosomal miRs isolated from the blood of 17 patients with NB revealed that 3779 miRs are differentially expressed in comparison with exosomal miRs from healthy controls. Among them, miR-199a-3p, which presence correlated with disease severity, was reported to increase proliferation and migration of NB cells in vitro [96]. A longitudinal analysis of exo-miRs expression profile in plasma samples from 52 children with high-risk NB obtained before and after induction chemotherapy performed by the International Society of Pediatric Oncology Europe Neuroblastoma (SIOPEN) revealed a three exo-miRs signature (miR-29c, miR-342-3p, and let-7b) that could discriminate between poor and good responders. Exo-miRs expression also provided a chemoresistance index predicting the good or poor prognosis of high-risk patients [97]. There is thus some evidence suggesting that the detection of exosomal miRs in the blood of patients with NB could be used as liquid biopies and biomarkers of aggressivity and response to therapy. A confirmation in larger numbers of patients is however needed.

## 5. Conclusions: Toward Genomic and TME Informed Clinical Trials

**Figure 2:**
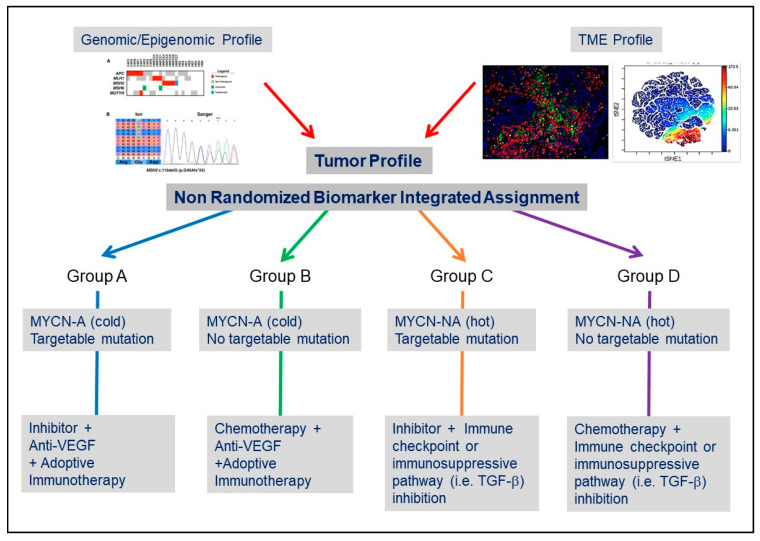
Considering the TME in the design of clinical trials in NB. Simplified conceptual design of a biomarker-integrated umbrella protocol in NB based on genomic and TME information.

The recent advances in our understanding of the complexity of the TME in NB, and the mechanisms of communication between NB cells and the TME summarized in this article, provide an opportunity to envision their potential impact on the treatment of NB as we progressively move from randomized clinical trials to more precise (or personalized) biomarker-integrated clinical trials [98]. Such precision medicine-based clinical trials have begun to be developed in NB. The Next-Generation Personalized Neuroblastoma Therapy (NEPENTHE Trial, NCT02780128) developed at Children’s Hospital of Philadelphia, is a phase 1 trial in which genomic aberrations in tumor cells at time of relapse are matched to rationally designed combinations of molecularly targeted agents. The Pediatric Precision Laboratory Advanced Neuroblastoma Therapy (PEDS-PLAN, NCT02559778) trial sponsored by the Beat NB Cancer Foundation and Spectrum Health Hospital is a prospective open-label, multicenter study to evaluate the feasibility and acute toxicity of using molecularly guided therapy in combination with standard therapy for children with newly diagnosed high-risk NB. It is also clear that trials solely guided by genomic and transcriptomic information may not be “precise” enough in particular, as new therapeutic approaches increasingly include antibody or cell-based immunotherapy [99,100]. In addition to the identification of drivers and targetable mutations, detailed information not only on the composition of the TME but also on the location of TME cells and on mechanisms of NB cell-TME cell interaction will have to complement genomic information. The inclusion of information on the TME combined with genomic information on the tumor cells is the objective of the Neuroblastoma Precision Trial (NCT02868268) developed by the New Approaches to Neuroblastoma Therapy (NANT) consortium. Longitudinal information on the TME landscape, immunoscore and immune monitoring will become an integral part of the evaluation of a tumor at the time of diagnosis, during therapy and at the time of recurrence [101]. Information on the status of the vasculature may provide a clue whether a tumor is “immune exclusive”, the presence or absence of stromal cells such as MSCs or CAFs or TAMs may point to immunosuppressive mechanisms associated with a high release of cytokines and chemokines. A spatial analysis of immune cells may suggest immune exclusion and alert to the limitations of an immune checkpoint blockade-based therapy or a cell-based therapeutic approach (Figure 2). For example, an Alk inhibitor in combination with a VEGF inhibitor and a cell-based therapy may be more suitable approach for a “cold” MYCN-A tumor exhibiting an Alk mutation and a dysfunctional vasculature. In contrast, a ‘hot” MYCN-NA tumor without targetable mutation may be better treated with a combination of chemotherapy and an immune checkpoint inhibitor or TGF-β inhibitor to prevent immune escape. 

We are thus at a critical and exciting point where our understanding of the contribution of the TME in NB will have a significant impact on our ability to increasingly personalize our treatments for this disease.

## Figures and Tables

**Table 1:** The TME Landscape in NB Tumors: Comparison between MYCN-A and MYCN-NA Tumors.

TME Cells		MYCN-A	MYCN-NA	Comments	Method	References
Stromal cells	Schwann cells (SC)	Rare	Variably present	SC-rich tumors rarely exhibit MYCN-A (2 over 19)	Histology	[33]
	Endothelial cells (EC)	Inc.	Present	MYCN-A tumors have a higher (>4.0) microvascular index	Histology	[34]
		Inc.	Dec.	MYCN-A have a higher expression of αvβ3 in their EC than MYCN-NA tumors (68 vs. 34%)	IHC	[35]
	Pericytes	ND	ND	No studies have examined pericytes in MYCN-A and MYCN-NA tumors		
	αSMA^+^ cells	Inc.	Present	Higher presence of non-pericytes αSMA expressing CAFs in MYCN-A vs. MYCN-NA tumors (*p* = 0.045)	IHC	[29]
	Osteoblasts, αSMA^+^ cells	Inc.	Present	Higher xCell score for mRNA from stromal cells (osteoblasts, smooth muscle cells) but not fibroblasts, MSC and EC in MYCN-A tumors	Transcriptomics in silico	[32]
Immune cells	Inflammatory cells	Dec.	Present	Decrease mRNA expression signature for CD8^+^T, CD4^+^T, B, macrophages, dendritic cells, and NK cells in MYCN-A tumors	Transcriptomics and IHC	[36]
	Inflammatory cells	Present	Inc.	Higher xCell score for mRNA from myeloid and lymphoid cells (DC, CD8^+^ T cells, B cells, NKT cells and macrophages) in MYCN-NA tumors	Transcriptomics in silico	[32]
	CTL	No difference	No difference	Presence of a subset of infiltrating T cells (high CD3^+^ or low CD3^+^ and high CD4^+^/ CD8^+^ ratio) in good prognosis MYCN-A tumors	IHC	[37]
	CTL	Dec.	Inc.	MYCN-NA tumors have significantly higher CTL signatures	RNA Seq, TMA, TCR sequencing	[38]
	Treg	Inc. in PB and BM	ND	*MYCN* amplification correlates to a higher number of Treg in BM and of Tr1 cells in PB	Flow cytometry on PB and BM	[32,39]
	B cells	Dec.	Present	B cells are rare or absent in MYCN-A tumors	RNA Seq. data in silico and IHC	[36]
	NKT and iNK T cells	Rare	Inc.	iNKT cells infiltrate NB with low MYCN-low and CCL2-high expression. NKT cells are more abundant in MYCN-NA tumors. Reverse correlation between iNKT cells genes and *MYCN* gene expression	Transcriptomics in silico; RT-PCR gene expression analysis; IHC	[24,32,36]
	NK	Dec.	Present	NK cells are decreased in MYCN-A tumors. There is an inverse correlation between MYCN expression and that of ligands for NK-cell-activating receptors	Transcriptomics and IHC	[36,40]
	M2 Macrophages,	ND	ND	CSF-1R^+^ myeloid cells predict poor clinical outcome. No comparison between MYCN-A and MYCN-NA done	Transcriptomics	[40]
	M2 macrophages	Present	Inc.	CD163^+^ macrophages are increased in MYCN-NA tumors	Gene expression array, TMA and IHC	[41]
	M2 macrophages	ND	Dec.	Higher presence of CD163^+^ macrophages in MYCN-A tumors	IHC	[29]
	Granulocytes	ND	ND	No evidence	ND	
	Basophil cells	Inc.	Present	MYCN-A groups show higher proportion than MYCN-NA tumors	Transcriptomics in silico	[32]

The table summarizes a review of the recent literature comparing MYCN-A and MYCN-NA tumors. ND = no data. Inc. = increase. Dec. = decreased.
